# Functional Characterization of the GATA-Type Transcription Factor PaNsdD in the Filamentous Fungus Podospora anserina and Its Interplay with the Sterigmatocystin Pathway

**DOI:** 10.1128/aem.02378-21

**Published:** 2022-03-22

**Authors:** Ling Shen, Thomas Gaslonde, Catherine Roullier, Huijuan Wang, Jennifer Bodin, François-Hugues Porée, Gwenaël Ruprich-Robert, Florence Chapeland-Leclerc

**Affiliations:** a Université de Paris, CNRS, Laboratoire Interdisciplinaire des Energies de Demain, Paris, France; b Shenzhen University, Shenzhen Key Laboratory of Microbial Genetic Engineering, College of Life Sciences and Oceanography, Shenzhen, China; c Université de Paris, CNRS, Cibles Thérapeutiques et Conception de Médicaments, Paris, France; d Université de Nantes, Mer Molécules Santé, Nantes, France; e Université de Rennes 1, Rennes Institute of Chemical Sciences, UMR 6226 CNRS, Laboratoire de Chimie Thérapeutique, Faculté de Pharmacie, Rennes, France; Nanjing Agricultural University

**Keywords:** transcription factor PaNsdD, sterigmatocystin, sexual development, 3-acetyl-4-methylpyrrole, *Podospora anserina*

## Abstract

The model ascomycete Podospora anserina, distinguished by its strict sexual development, is a prolific but yet unexploited reservoir of natural products. The GATA-type transcription factor NsdD has been characterized by the role in balancing asexual and sexual reproduction and governing secondary metabolism in filamentous fungi. In the present study, we functionally investigated the NsdD ortholog PaNsdD in P. anserina. Compared to the wild-type strain, vegetative growth, ageing processes, sexual reproduction, stress tolerance, and interspecific confrontations in the mutant were drastically impaired, owing to the loss of function of PaNsdD. In addition, the production of 3-acetyl-4-methylpyrrole, a new metabolite identified in P. anserina in this study, was significantly inhibited in the *ΔPaNsdD* mutant. We also demonstrated the interplay of PaNsdD with the sterigmatocystin biosynthetic gene pathway, especially as the deletion of *PaNsdD* triggered the enhanced red-pink pigment biosynthesis that occurs only in the presence of the core polyketide synthase-encoding gene *PaStcA* of the sterigmatocystin pathway. Taken together, these results contribute to a better understanding of the global regulation mediated by PaNsdD in P. anserina, especially with regard to its unexpected involvement in the fungal ageing process and its interplay with the sterigmatocystin pathway.

**IMPORTANCE** Fungal transcription factors play an essential role in coordinating multiple physiological processes. However, little is known about the functional characterization of transcription factors in the filamentous fungus Podospora anserina. In this study, a GATA-type regulator PaNsdD was investigated in P. anserina. The results showed that PaNsdD was a key factor that can control the fungal ageing process, vegetative growth, pigmentation, stress response, and interspecific confrontations and positively regulate the production of 3-acetyl-4-methylpyrrole. Meanwhile, a molecular interaction was implied between PaNsdD and the sterigmatocystin pathway. Overall, loss of function of PaNsdD seems to be highly disadvantageous for P. anserina, which relies on pure sexual reproduction in a limited life span. Therefore, PaNsdD is clearly indispensable for the survival and propagation of P. anserina in its complex ecological niches.

## INTRODUCTION

The model fungus Podospora anserina, recently described as Triangularia anserina (Sordariales, Ascomycota) (1), has long been used in the laboratory to study various biological processes, such as senescence, meiosis, prions, sexual development, signal transduction, cell fusion, hyphal interference, vegetative incompatibility, mitochondrial physiology, and plant biomass degradation (2–5). P. anserina is frequently recovered from herbivore dung and is therefore regarded as a typically coprophilous fungus (4, 6). Dung-inhabiting fungi are predominately known to produce various secondary metabolites that act as chemical weapons to enhance competitiveness and ecological fitness (7, 8). These bioactive products are of intense interest to humankind because of their potential pharmaceutical properties (9, 10). Notably, mining of the P. anserina genome revealed a large number of putative biosynthetic gene clusters (BGCs) for secondary metabolites (11). However, to date, only very few products that exhibited antibacterial, antifungal, and larvicidal activities have been characterized in this species during past decades (12–14), probably because most BGCs are silent under standard culture conditions (10, 15). Genomics-driven BGC detection and preliminary chemical investigations so far imply that P. anserina is a prolific, as-yet unexploited natural product reservoir.

In fungi, secondary metabolism is often governed by intricate regulatory mechanisms that also control other processes (e.g., asexual and sexual development) (16, 17). For example, the global transcription factor LaeA (loss of *aflR* expression) simultaneously regulates numerous metabolic BGCs and fungal morphogenesis in Aspergillus spp., as well as in Fusarium verticillioides (Hypocreales, Ascomycota), Monascus pilosus (Eurotiales, Ascomycota), and Penicillium dipodomyis (Eurotiales, Ascomycota) (18–22). As core regulatory elements, transcription factors indeed play an essential role in multilevel gene expression coordination (23). Our previous work characterized the mycotoxin sterigmatocystin (ST) pathway-specific transcription factor PaAflR, which mediates biological processes, including metabolite production, sexual development, pigmentation, and interspecific competition in P. anserina (14). Nevertheless, plenty of transcription factors in P. anserina still remain to be elucidated (24).

The *NsdD* gene (never undergo sexual development) encodes a GATA-type zinc finger transcription factor, with highly conserved DNA-binding domain in many filamentous fungi but not in plants or animals (25). NsdD was initially identified as a key activator of sexual reproduction in Aspergillus nidulans (Eurotiales, Ascomycota) due to the complete loss of fertility in the corresponding mutant (25). From then on, NsdD and its orthologs (SUB-1/Pro44/Ltf1/Csm1/Nsd1) were characterized according to their involvements in biological processes in filamentous fungi. The roles of NsdD and its orthologs are summarized in Table 1. For example, NsdD affected morphogenesis and aflatoxin (AF) production in Aspergillus flavus (Eurotiales, Ascomycota) (26) and was identified as a repressor of asexual development and negatively affected ST production in A. nidulans (27). Deletion of *Pro44* caused developmental defects and sterility in Sordaria macrospora (Sordariales, Ascomycota) (28). Deletion of *Csm1* resulted in enhanced microconidia formation, reduced stress tolerance, and deregulated expression of BGCs in Fusarium fujikuroi (Hypocreales, Ascomycota) (29). Loss of SsNsd1 function affected morphological transition, appressoria formation, and pathogenicity in the plant pathogen Sclerotinia sclerotiorum (Helotiales, Ascomycota) (30). However, the role of NsdD in the model fungus P. anserina, which lacks asexual reproduction, has not been studied.

**TABLE 1:** Role of NsdD and orthologs in some filamentous fungi

Fungi	Protein	Role	Reference
Aspergillus nidulans	NsdD	Fertility and sterigmatocystin production	25, 27
Neurospora crassa	SUB-1	Perithecia production	56
Aspergillus flavus	NsdD	Morphogenesis and production of aflatoxin	26
Sordaria macrospora	Pro44	Developmental cycle and fertility	28
Trichoderma reesei	SUB1	Regulation in a nutrient- and light-dependent manner of female fertility, pheromone system, and secondary metabolism	36
Botrytis cinerea	BcLtf1	Regulation of light-dependent stress responses and expression of secondary metabolism-related genes	45
Fusarium fujikuroi	Csm1	Microconidia formation, stress tolerance, and expression of biosynthetic gene clusters	29
Sclerotinia sclerotinium	SsNsd1	Morphological transition, appressoria formation, and pathogenicity	30
Penicillium oxalicum	PoxNsdD	Regulation of major genes involved in plant biomass degradation, conidiation, and pigment biosynthesis	37
Metarhizium rileyi	MrNsdD	Dimorphic transition and microsclerotia formation	46
Podospora anserina	PaNsdD	Vegetative growth, sexual reproduction, stress tolerance, interspecific confrontation, and production of 3-acetyl-4-methylpyrrole	This study

In the present study, we functionally investigated the global regulator PaNsdD in P. anserina. Our results revealed that PaNsdD was required for vegetative growth, sexual reproduction, stress tolerance, interspecific confrontation, and production of 3-acetyl-4-methylpyrrole, a new secondary metabolite identified in P. anserina in this study. We also demonstrated the interplay of PaNsdD with the ST pathway. Indeed, the deletion of *PaNsdD* triggers the enhanced red-pink pigment biosynthesis in the presence of the core polyketide synthase (PKS)-encoding gene *PaStcA* of the ST pathway.

## RESULTS

### Identification of *PaNsdD* and generation of mutants.

In P. anserina, a total of 507 transcription factors classified into 41 families can be retrieved from the Fungal Transcription Factor Database (24). Among them, the P. anserina genome contains a putative *NsdD* ortholog, namely *PaNsdD* (*Pa_2_1880*). The complete CDS of *PaNsdD* consists of 1,659 bp and potentially encodes a GATA-type transcription factor, namely PaNsdD, of 411 amino acids. PaNsdD shares 99.32, 57.43, and 56.57% identities with NsdD orthologs from Podospora comata, Neurospora crassa, and S. macrospora, respectively. In order to study the biological functions of PaNsdD in P. anserina, a deletion mutant lacking *PaNsdD* (*ΔPaNsdD*) and complemented strain (*ΔPaNsdD^C^*) were constructed, verified (Fig. S1), and subjected to further analysis.

### Impact of PaNsdD on vegetative growth and colony morphology.

**FIG 1:**
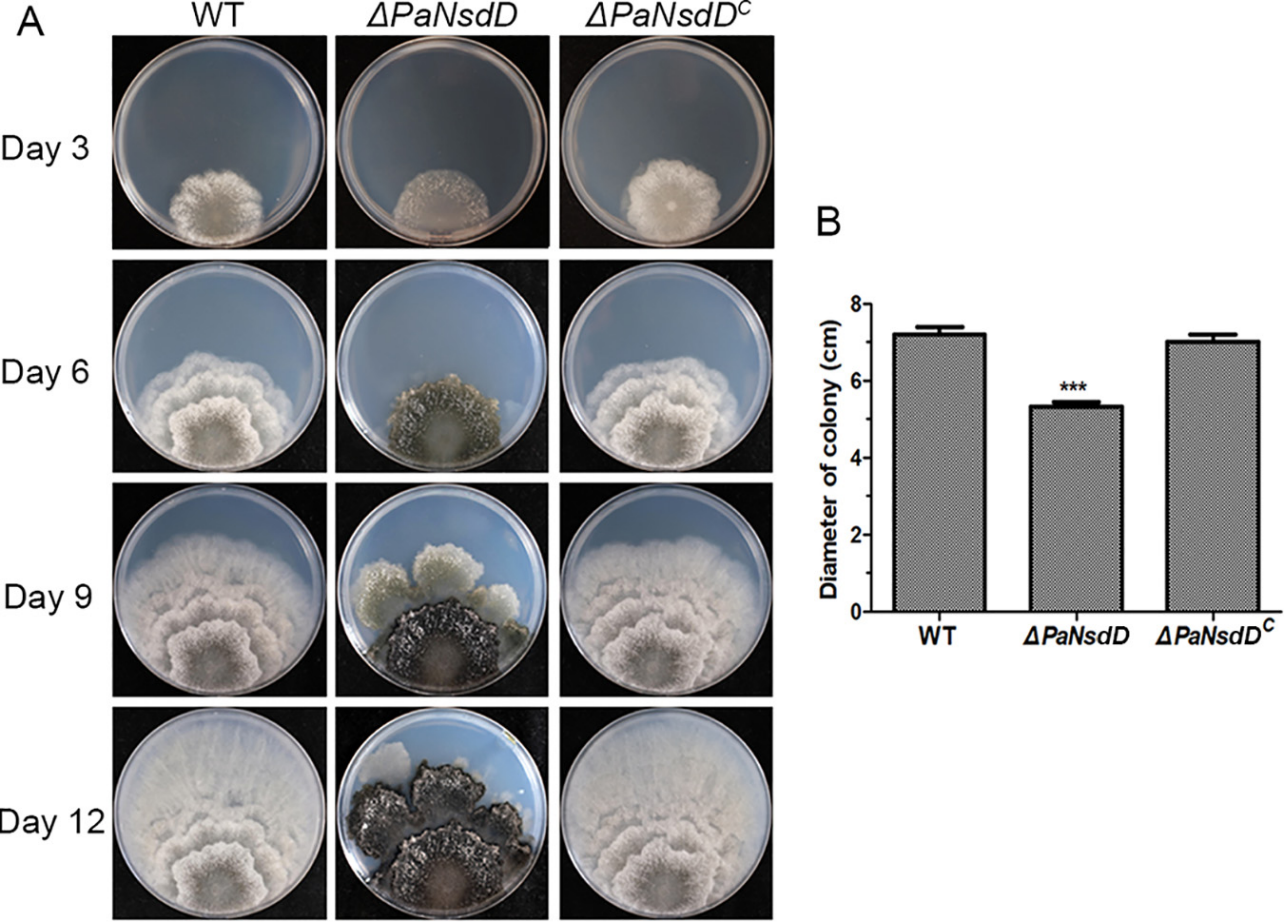
Colony morphology of P. anserina strains. (A) The wild-type (WT), *ΔPaNsdD*, and *ΔPaNsdD^C^* strains were grown on M2 solid medium at 27°C under dark conditions. The pictures were taken after several days of incubation as indicated. Inoculum was positioned on the edge of the petri dish in order to observe the growth of the strains on a large surface, necessary for 12 days of growth. (B) Colony diameter of tested strains after 5 days of growth. As the inoculation point was made on the edge of the plate, the radius of the colony was measured, the diameter is then deduced by multiplying this length by two. The data represent the means ± standard deviation (SD) from three independent experiments. Asterisks indicate significant differences (*P < *0.01) of each strain relative to the WT, followed by Student’s *t* test.

To evaluate the impact of PaNsdD on vegetative growth and colony morphogenesis, wild-type and mutant strains were cultured on M2 solid medium for 12 days. In the first stage, the *ΔPaNsdD* mutant displayed a reduction in growth rate compared to the wild-type strain (Fig. 1). Moreover, the *ΔPaNsdD* strain formed a flat colony due to sparse aerial hyphae formation. Intriguingly, in the second stage from the 6th day to the 9th day, growth of *ΔPaNsdD* appeared to suddenly cease since culture began to slow down, and hyphal prolongation at the edge of the colony was clearly blocked to a large extent. Meanwhile, accelerated and excessive accumulation of pigment started to appear on the surface of colony in a short period, usually less than 3 days (Fig. 1). Nevertheless, the remaining hyphae that were not arrested in partial section could escape the front of growth and persist with outward extension. In the third stage from the 9th to the 12th day, active hyphae that went beyond the colony margin restarted a series of processes, including vegetative proliferation, cessation, and melanization (Fig. 1). It should be noted that more and more mycelia were gradually arrested during this periodic process; only some hyphae can avoid the melanized fate and continue their weak growth until final growth cessation. As shown in Fig. 1, deletion of *PaNsdD* eventually led to the irregular peripheral growth, accompanied by hyphal staling and enhanced pigmentation. In contrast, the *ΔPaNsdD^C^* strain showed wild-type-like growth characteristics, such as normal growth rate, fluffy colony with extensive aerial hyphae, and normal pigmentation. These results indicated that PaNsdD was crucially required for the correct growth and morphogenesis in P. anserina; otherwise, a cycled stop-start growth pattern might be triggered, leading to an affected ageing process.

### Role of PaNsdD in sexual reproduction.

**FIG 2:**
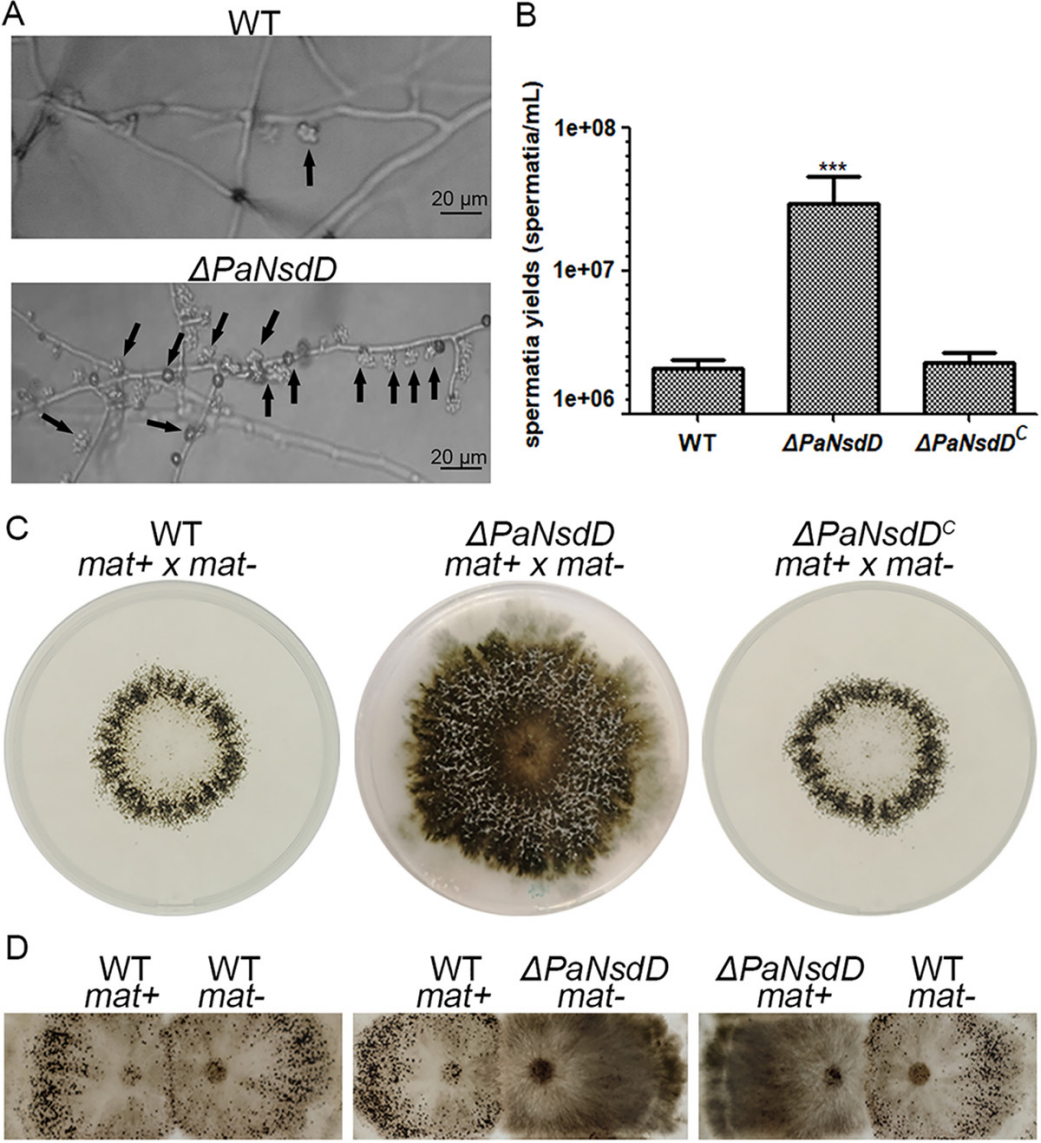
Sexual development of P. anserina strains. (A) Microscopic observation of spermatia formation. The pictures were taken after 3 days of growth on M2 plate. The arrows indicate typical spermatia. (B) Quantification analysis of spermatia. The tested strains were cultured on M2 medium for 3 days and then used for the counting and observation of spermatia. The data represent the means ± SD from three independent experiments. Asterisks indicate significant differences (*P < *0.01) of each strain relative to the WT, followed by Student’s *t* test. (C) Fertility assays. Fragmented mycelia from opposite mating type strains were mixed and then inoculated in the center of plate. The plates were grown at 27°C under constant illumination. The photos were taken after 8 days of incubation. (D) Spreading experiments. Crosses were made by inoculating both strains 1.5 cm apart. After 3 days of incubation, 1.5 mL of water was added and spread all over the plate. Mature perithecia were visible as small dots when the fertilization event was completed.

To study the role of PaNsdD in sexual development, we first examined the formation of reproductive organs through microscopic observation. Both opposite mating type strains (*mat+* and *mat*−) of P. anserina were able to differentiate male gametes (spermatia) and female gametes (ascogonia). Obviously, the *ΔPaNsdD* mutant exhibited enhanced production of spermatia on M2 solid medium following 3 days of cultivation (Fig. 2A). Subsequently, quantification analysis demonstrated that spermatia yield of *ΔPaNsdD* was significantly increased (4.5-fold, *P < *0.01), compared with wild-type strain (Fig. 2B). The complemented strain restored normal spermatia production. Therefore, these data suggested PaNsdD functions as a negative regulator of spermatia differentiation in P. anserina.

In view of the aberrant formation of spermatia caused by *PaNsdD* deletion, we further determined the fertility of wild-type and mutant strains. As expected, the wild-type strain produced a ring of perithecia after a fertilization event. In contrast, loss of PaNsdD led to the radial growth, but no perithecia were produced even if it was cultured for up to 1 month. Genetically complemented strain regained the wild-type-like fertility (Fig. 2C). This result indicated that the lack of PaNsdD completely abolished sexual reproductive ability. Due to the sterile characteristic, we then dissected the potential defects that could be present in the process of gamete differentiation, fertilization, or perithecial maturation. We showed that the *ΔPaNsdD* mutant acts as a maternal partner that failed to be fertilized by the spermatia of the wild-type strain, which acts as paternal partner (Fig. 2D). It implies that the absence of PaNsdD triggered potential defects in ascogonia. We finally confirmed that the female sterile phenotype in *ΔPaNsdD* was not dependent on mating type by reciprocal crosses. Taken together, largely consistent with the initial nomenclature, the data above revealed that the *PaNsdD* mutant indeed never undergo sexual development, which may be mainly due to the overproduction of spermatia and the dysfunction of ascogonia.

### Function of PaNsdD in multiple stress responses.

**FIG 3:**
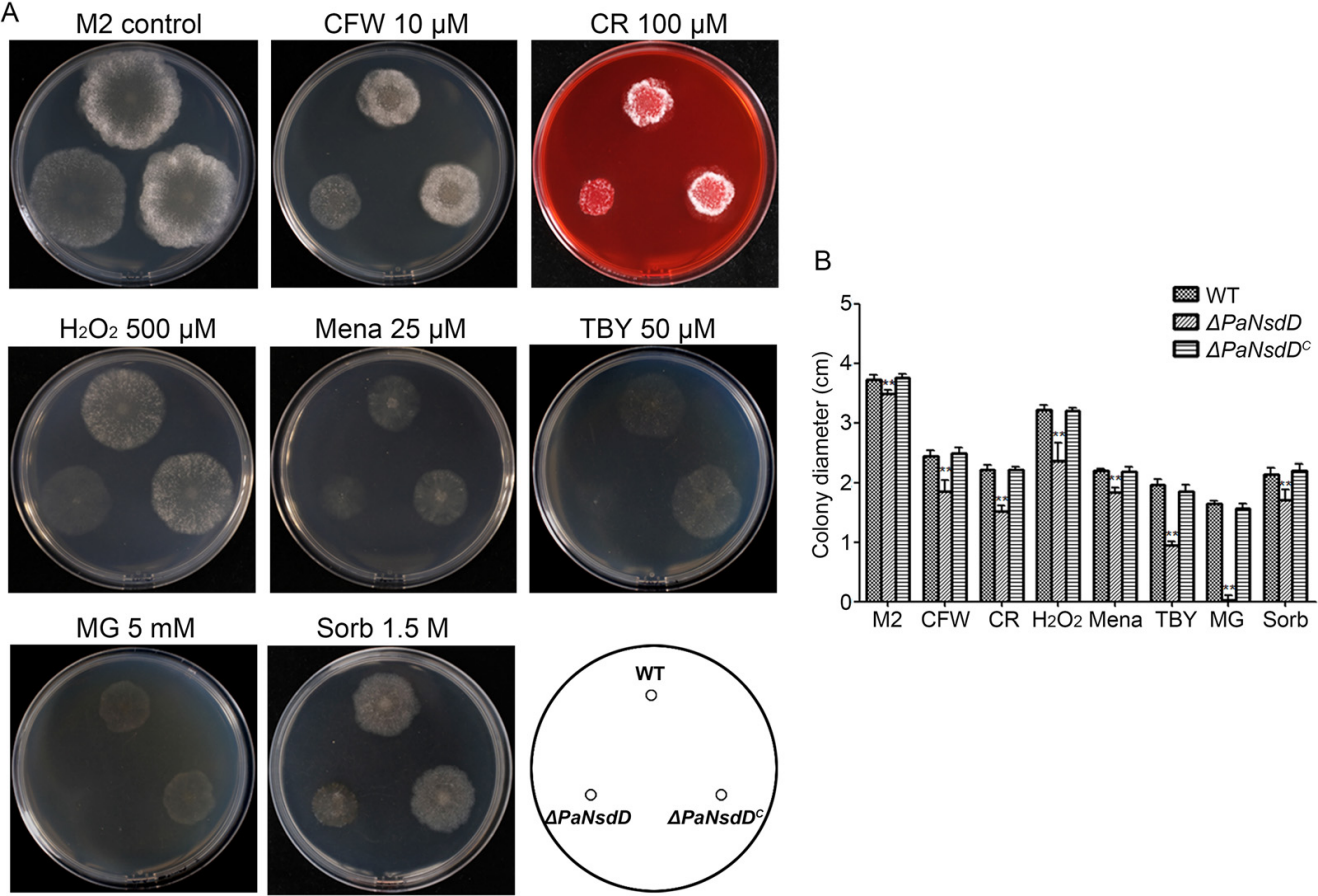
Growth of P. anserina strains on M2 plates exposed to abiotic stressors. The tested strains were grown on M2 medium supplemented with different stress agents under certain concentrations as indicated. (A) Colony morphology in the presence of stress-inducing agents. The pictures were taken after 3 days of incubation at 27°C. (B) Colony diameter (cm) of tested strains after 3 days of incubation at 27°C. The corresponding strains that grown on M2 medium without supplements were used for normalization. CFW, Calcofluor white; CR, Congo red; Mena, menadione; TBY, *t*-butyl hydroxyperoxide; MG, methylglyoxal; Sorb, sorbitol.

To clarify the function of PaNsdD in response to various stressors, we determined the fungal growth on M2 plates supplemented with stress-inducing agents. Strikingly, compared to the wild-type strain, the *ΔPaNsdD* mutant showed significantly reduced tolerance to different oxidative stressors, as H_2_O_2_ (500 μM), menadione (Mena, 25 μM), and *t*-butyl hydroxyperoxide (TBY, 50 μM) (Fig. 3). Especially, the growth of *ΔPaNsdD* was almost completely inhibited in the presence of methylglyoxal (MG) at 5 mM, which can induce reactive oxygen species (ROS) production by depleting intracellular glutathione (31). Moreover, growth of *ΔPaNsdD* was highly affected in the presence of sorbitol at 1.5 M. Additionally, to a different extent, growth of mutants was also inhibited under cell wall stress conditions triggered by exposure to Congo red (CR, 100 μM) and Calcofluor white (CFW 10 μM), which impair assembly of β-1,4-glucans and chitin in the cell wall, respectively (Fig. 3). Taken together, increased sensitivity of *ΔPaNsdD* to various stressors indicated that PaNsdD was required for the maintenance of stress tolerance and cell wall integrity in P. anserina.

### Contribution of PaNsdD in interspecific confrontations.

**FIG 4:**
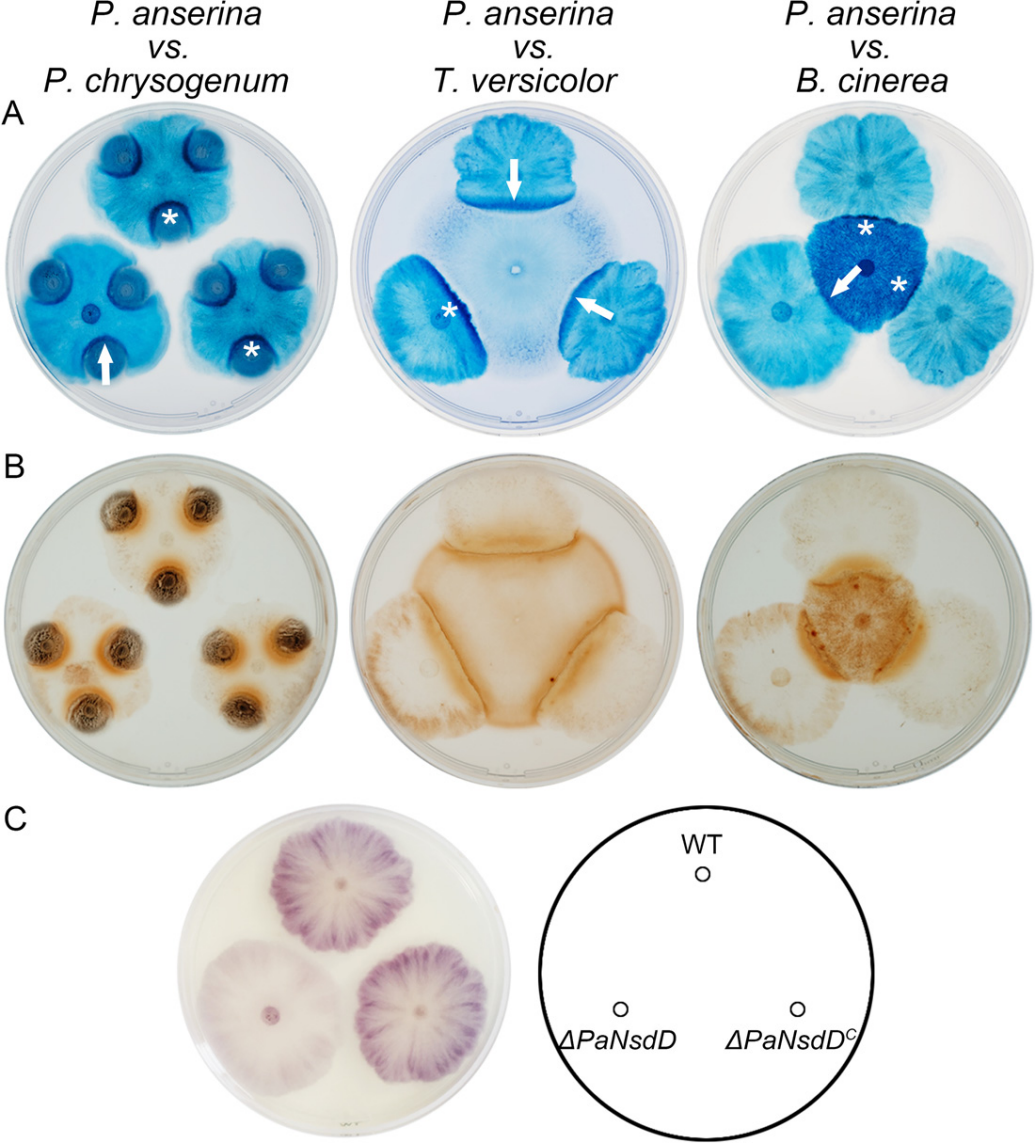
Confrontation of P. anserina strains against other fungal species. Cell death assay (A), peroxide detection (B), and superoxide detection (C) were conducted as previously described (14, 32). (A) In the contact zone, the intensity difference of blue color indicates the Trypan blue-stained dead cells triggered by the contestant strains. Asterisks indicate more serious cell death than that indicated by arrows. (B) Peroxide detection of P. anserina strains when contestants were involved. (C) Superoxide detection in WT, *ΔPaNsdD*, and *ΔPaNsdD*^C^ in the absence of challengers.

To elucidate the contribution of PaNsdD in interspecific interactions, the *ΔPaNsdD* mutant and the wild-type strain were coconfronted with three other fungal challengers, as previously described (14, 32). In the cell death assay, we found that Penicillium chrysogenum (Eurotiales, Ascomycota) and Botrytis cinerea that have been described to be usually killed by P. anserina displayed a lower level of cell death in the contact zone when confronted to *ΔPaNsdD* compared to wild type (Fig. 4A). In contrast, Trametes versicolor (Polyporales, Basidiomycota), which usually can kill P. anserina (14, 32), aggravated cell death specifically on the thallus of *ΔPaNsdD*, compared to confrontation with wild type. These results implied that loss of PaNsdD triggered the declining competitiveness of P. anserina during interspecific antagonistic processes. Then, we detected the level of peroxide, which is usually restricted to the contact zone, and superoxide, that is accumulated more or less uniformly on fungal thallus, without any necessary contact with the competitor (32). No obvious difference in peroxide accumulation is observed between wild type and *ΔPaNsdD* at the confrontation with contestant strains (Fig. 4B). Moreover, peroxide accumulation was not detected on wild-type and mutant thalli, in the absence of competitor (Fig. S2). Finally, nitroblue tetrazolium (NBT) detection assay revealed that *ΔPaNsdD* produced a lower level of superoxide over the thallus than that of the wild-type strain (Fig. 4C), indicating that PaNsdD was probably involved in the production of ROS, mainly superoxide. Combined with the decreased tolerance of *ΔPaNsdD* to abiotic stresses, we suggested that PaNsdD might contribute to a positive role in protecting P. anserina from hostile environment by mediating ROS production.

### Effects on fungal pigmentation and interplay of PaNsdD with the ST pathway.

**FIG 5:**
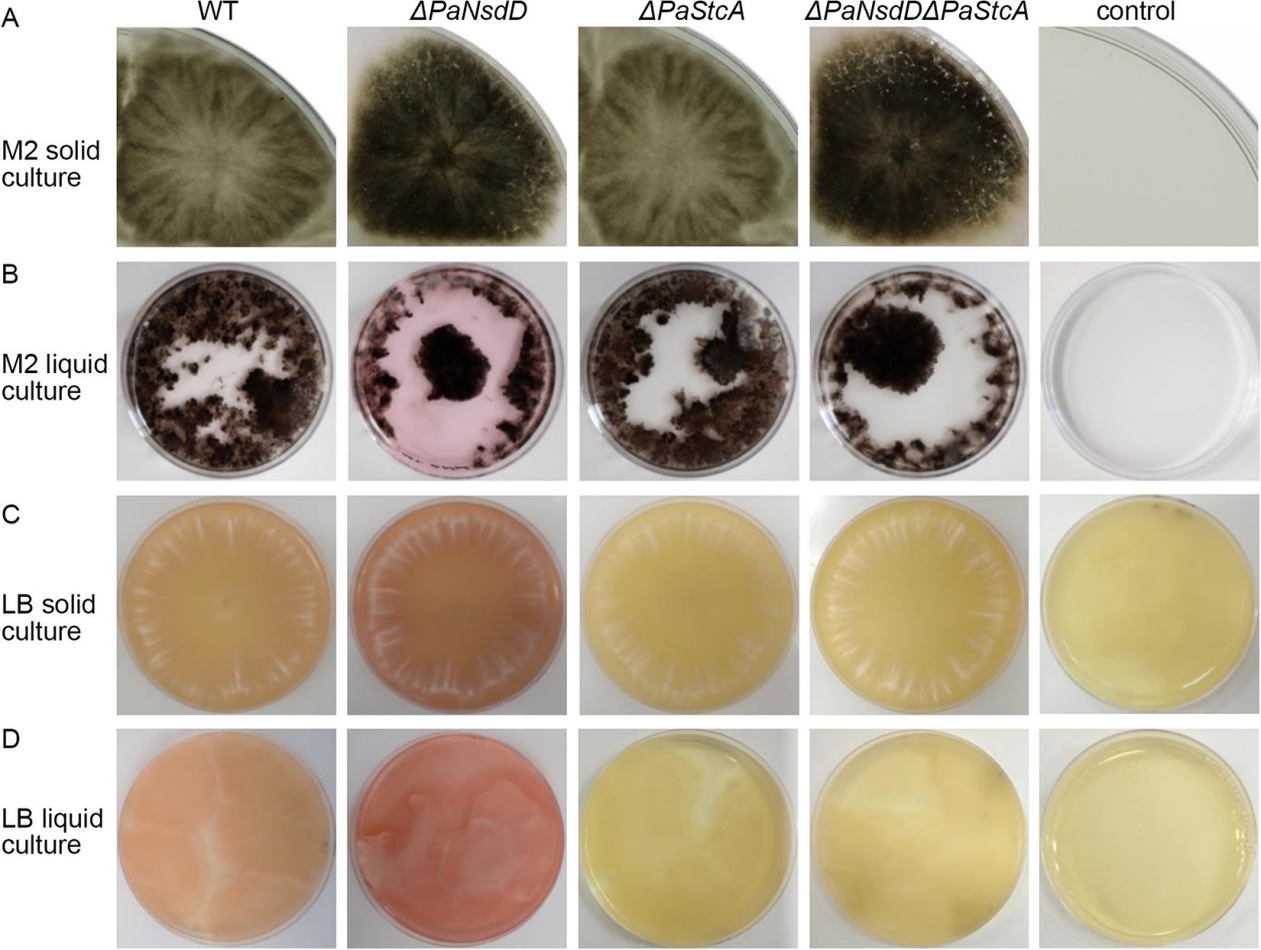
Pigmentation of P. anserina on various media. Tested strains were cultured on M2 solid medium (A), M2 liquid medium (B), LB solid medium (C) and LB liquid medium (D), respectively. After 14 days of growth at 27°C, altered pigmentation were clearly observed, and photos were therefore taken.

To investigate the impact of PaNsdD on fungal pigmentation, all of the strains were cultured on M2 and LB media, liquid and solid in both cases. As shown in Fig. 5, P. anserina wild-type strain was normally greenish on M2 medium, due to accumulation of melanin, and presented a slight red-pink pigmentation in M2 liquid medium and on LB plate. Nevertheless, we first observed that the coloration is more intense under all conditions with *ΔPaNsdD* compared to wild type, suggesting an excessive accumulation of pigments in this mutant. Then, one might expect that PaNsdD could downregulate the pigment production or affect its accumulation and could be involved in the melanin biosynthesis in particular. It should be noted here that when strains were grown on G medium and G + yeast extract medium (culture media usually used to promote ascospore germination), the mycelium remained everywhere very poorly pigmented, and the culture medium remained unstained (Fig. S3). Taken together, all these results are in accordance with the fact that fungal coloration highly depends on the medium composition (5).

Nevertheless, in a previous work, we observed (i) that the absence of the red-pink pigmentation when the *ΔPaStcA* mutant lacking the ST-core polyketide synthase PaStcA was cultured on M2 and (ii) the accumulation of red-pink pigments in the ST-overproducing strain OE-PaAflR (14), which seem to be identical to those produced by the *ΔPaNsdD* strain. Similar results were obtained here on M2 but also on LB medium (Fig. 5B to D). In order to better understand the putative interplay of PaNsdD with the ST pathway and the occurrence of a similar red-pink pigmentation phenotype, the double mutant *ΔPaNsdDΔPaStcA* was constructed.

This mutant exhibited a colorless colony phenotype identical to *ΔPaStcA* on M2 liquid and LB media (Fig. 5B to D). Therefore, these results suggested that the presence of PaStcA was a prerequisite for the biosynthesis of red-pink pigments and that this gene interplays with pigment production. Our findings here are consistent with those previously described; i.e., that such a red-pink pigmentation requires the presence of functional ST pathway (14). It is worth noting that cultures of *ΔPaStcA* were always colorless even when they were subjected to 1 month of incubation under the same conditions (data not shown).

### Involvement of PaNsdD in the biosynthesis of a new metabolite in P. anserina.

**FIG 6:**
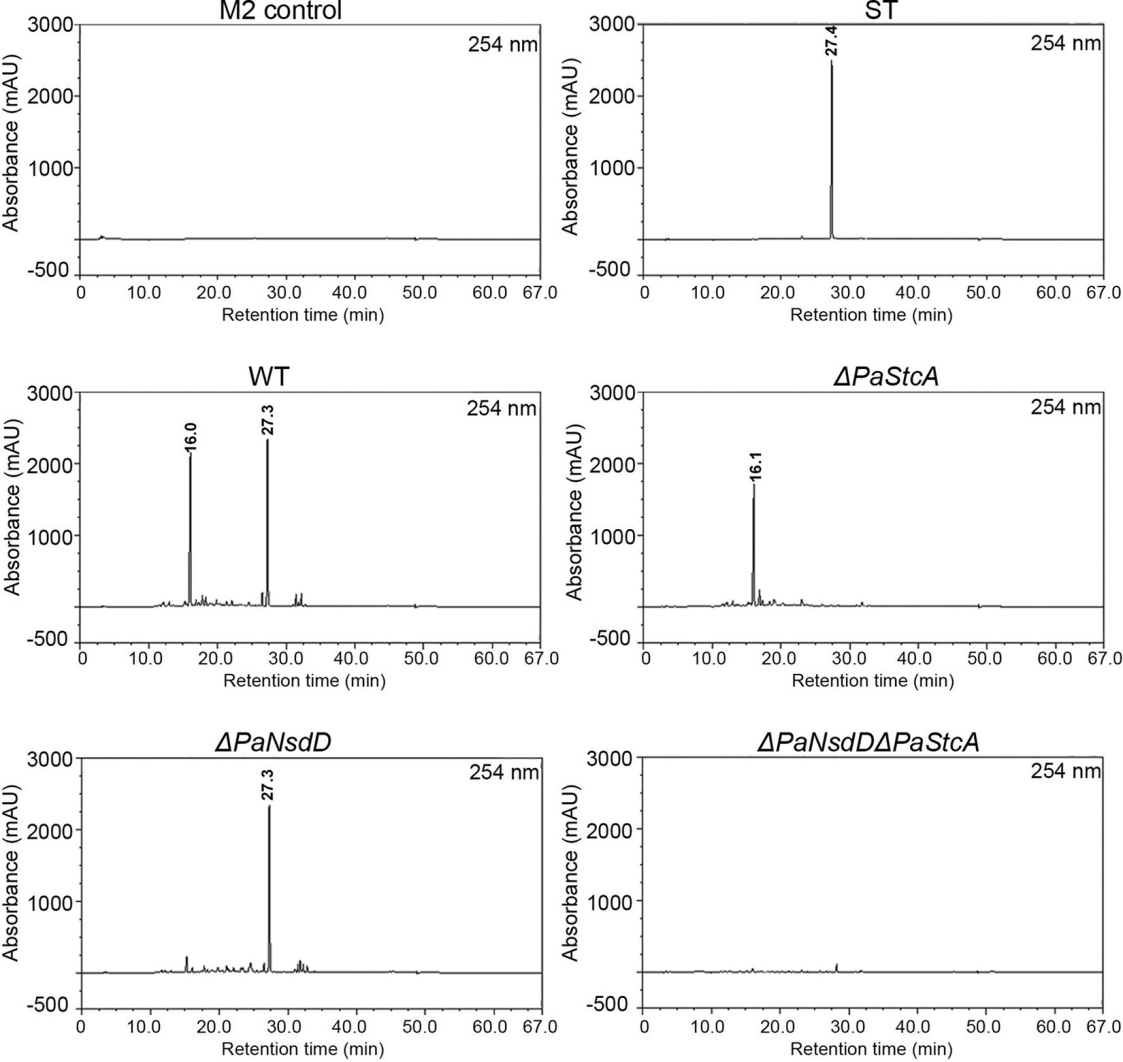
High-performance liquid chromatography UV (HPLC-UV) detection of culture extracts of P. anserina. The HPLC-UV profiling displayed the peak intensity (mAU) according to the retention time (rt). Sterigmatocystin (ST) presents a rt of 27.3 min (14). The other major peak presented a rt of 16.05 min. The *x* axis (min) shows the retention time. The *y* axis (mAU) shows the signal intensity.

To examine secondary metabolites regulated by the global transcription factor PaNsdD in P. anserina, we first conducted a qualitative and semiquantitative analysis by high-performance liquid chromatography UV (HPLC-UV) analysis in all extracts after 14-day cultivation, as previously described (14). We found that the secondary metabolite profile was significantly altered owing to the *PaNsdD* mutation. First, under M2 liquid condition, the profile of wild-type strain evidenced two major peaks at 16.05 and 27.3 min, respectively (Fig. 6). The second peak at 27.3 min could unambiguously be correlated with ST, as previously seen (14). We may note that the HPLC-UV profiling on LB culture extracts showed only one peak, clearly correlated with ST, in wild-type and *ΔPaNsdD* strains, meaning that the second compound is absent under these culture conditions (Fig. S4).

In order to identify the compound corresponding to the peak at 16.05 min, it was necessary to proceed to its isolation by silica chromatography and preparative HPLC successively. Characterization of this isolated product was conducted by liquid chromatography-mass spectrometry (LC-MS) in electron spray ionization (ESI)-positive and -negative modes. Our results showed that the corresponding signals of this product are two ions at *m*/*z* 124 for [M+H]^+^ and *m*/*z* 122 for [M − H]^−^, respectively (Fig. S5), indicating that the molecular formula of this compound could be C_7_H_9_NO. ^1^H and ^13^C nuclear magnetic resonance (NMR) along with 2D spectra (Fig. S6) allowed identification of 3-acetyl-4-methylpyrrole for the compound at 16.05 min. All of these data are in accordance with the literature (33–35). Thus, deletion of *PaNsdD* in both wild-type and *ΔPaStcA* background almost completely blocked the production of 3-acetyl-4-methylpyrrole (at 16.05 min), implying that PaNsdD positively regulates its biosynthesis. In contrast, the amount of ST, determined by the UV signal intensity (mAU), was similar in both wild type and *ΔPaNsdD*, indicating that the production of ST was not affected by the deletion of *PaNsdD* (Fig. 6).

## DISCUSSION

### PaNsdD is an activator of sexual reproduction and a repressor of male gamete production in P. anserina.

Given the critical role of NsdD in balancing sexual and asexual development in several fungi (29, 30, 36–38), we wondered about the function of PaNsdD in P. anserina, which has only a pure sexual life cycle without any asexual phase (4). Our data suggested that PaNsdD is a positive regulator of sexual reproduction and a repressor of male gametes production in P. anserina. Unlike other asexual spores, spermatia produced by P. anserina were only used to fertilize ascogonia and had almost no germination (38). Here, we show that the erected sterile hyphae of *ΔPaNsdD* mutant was able to form anamorphic-like structures containing dozens of spermatia, which strikingly resemble those of Cladorrhinum*/*Bahupaathra species (5).

### PaNsdD is involved in the vegetative growth pattern of P. anserina.

Remarkably, *ΔPaNsdD* mutant was distinguished by an unusual growth characteristic that involved an alternation of irregular periods of growth and nongrowth on M2 solid medium. In this study, we presented the infrequent alternative growth pattern in P. anserina, however, that resembled the ones observed in Neurospora and Aspergillus species. For instance, unlike the continuous outward extension of hyphae in the wild-type strain, the “stopper” mutants of N. crassa had an aberrant phenotype in which growth of the mycelium started, stopped, and then started again (39). The “vegetative death” mutants of Aspergillus glaucus (Eurotiales, Ascomycota) showed many features of senescence similar to those described for P. anserina. However, a partial section of A. glaucus mutant culture sometimes can escape the dying front of mycelia and continue the patchy extension until ultimate growth cessation (40). Taken together, compared to the wild-type strain of P. anserina, the *ΔPaNsdD* mutant showed persistent cycles of stopping and impaired starting, with a concomitant excessive accumulation of pigment, leading to a clear disturbance in the ageing process of the thallus. As we know, the ageing model P. anserina clearly did not propagate indefinitely and underwent senescence during its limited vegetative growth period (4, 41). In P. anserina, strain-specific senescent phenotypes were characterized by some common features, such as darker pigmentation, growth stop at the hyphal tips, and formation of “barrier of senescence” (5). Noticeably, visible morphological changes in *ΔPaNsdD* mutant, such as sparse aerial hyphae, overproduction of dark pigment, accelerated cessation of hyphal growth, rapid transition from juvenile phase to senescent phase, and ragged elongation of partial peripheral hyphae, indicated the premature senescence syndrome caused by *PaNsdD* mutation.

### PaNsdD is required for fungal pigmentation and stress tolerance.

Pigmentation is generally considered the output of secondary protective mechanisms that protect the producer from adverse environmental stresses, including oxidative stress, light, UV radiation, and temperature variation (42–44). Moreover, given the essential role of NsdD and its orthologs in maintaining the redox homoeostasis in other fungi (29, 45), we hypothesized that the overpigmented *ΔPaNsdD* mutant would respond to multiple stressors. However, we found that *ΔPaNsdD* mutant was hypersensitive in the presence of various oxidant and osmotic stressors, suggesting that here, enhanced accumulation of pigment did not confer a protective function onto P. anserina. Moreover, a likely scenario could be that defects in cell morphogenesis might lead to internal stress signals caused by aberrant cell wall formation, which in turn could be the reason for the observed sensitivity toward several stresses or the enhanced melanization. Our findings were in accordance with the functions of BcLtf1 in B. cinerea and MrNsdD in Metarhizium releyi in mediating ROS homoeostasis and stress response (45, 46).

### PaNsdD affects secondary metabolism in P. anserina.

Apart from the altered growth pattern and differentiation affected by the loss of function of PaNsdD, pigment variation of mutant cultures in both solid and liquid medium indicated that PaNsdD played a vital role in governing some secondary metabolism pathways in P. anserina. Indeed, previous studies revealed that NsdD and its orthologs function as global regulators in secondary metabolism processes in several fungi, and in particular, NsdD influenced ST biosynthesis in A. nidulans (27). In this study, we have highlighted a dark green pigment and a red-pink pigment that are overproduced in *ΔPaNsdD*. As the DHN-melanin has been described to be the major pigment that contributes to the greenish colony morphology of P. anserina in standard conditions (47), we hypothesize here that the dark green pigment is linked to the biosynthesis of melanin, but further experiments are necessary to clarify this point. Intriguingly, deletion of *PaNsdD* in both wild-type and *ΔPaStcA* backgrounds triggered a very dark pigmentation of the whole thalli, which is probably also linked to an excessive accumulation of melanin. We also found that the single mutant *ΔPaStcA* and the double mutant *ΔPaNsdDΔPaStcA* lost their abilities to produce the red-pink pigment, which is visible in both wild-type and *ΔPaNsdD* strains on M2 liquid medium and on LB plates, implying that the *ΔPaStcA* phenotype predominated in the double mutant under these conditions. In contrast, it seems that growth of P. anserina on LB medium was not able to induce fungal melanin production but led to the formation of a red-pink pigment, indicating that at least two pigment biosynthetic pathways exist in P. anserina. We do not know yet which chemical compound is responsible for the pigmentation, but we have demonstrated here that the presence of PaStcA was a prerequisite for the biosynthesis of this red-pink pigment and that PaNsdD negatively regulated the two pigmentation pathways.

Finally, secondary metabolites may serve as defense mechanisms during fungal colonization and the adaptation processes (48, 49). The interspecific competitiveness of *ΔPaNsdD* was suppressed when confronted with other fungal antagonists. Accordingly, in addition to the reduction of ROS production, the role of the new compound, identified here as 3-acetyl-4-methylpyrrole, needs to be further investigated. To this day, this compound was identified only in Eupenicillium hirayamae (Eurotiales, Ascomycota) under the name of desoxyverrucarin E (35) and in Aspergillus terreus (Eurotiales, Ascomycota) (34), but its involvement in fungal physiology has not yet been investigated.

To conclude, in this study, we demonstrated that deletion of *PaNsdD* led to a huge change in vegetative growth, pigmentation, stress response, and secondary metabolism regulation and that a molecular interaction was observed between PaNsdD and PaStcA. Overall, the loss of PaNsdD leads to premature ageing, sterility, and sensitivity to environmental stresses. Such features seemed to be highly disadvantageous for this species whose propagation before senescence and death exclusively relies on sexual reproduction. The presence of PaNsdD is clearly indispensable for the survival and propagation of P. anserina in its complex ecological niches.

## MATERIALS AND METHODS

### Strains and culture conditions.

The P. anserina strains used in this study were derived from the “S” (big S) wild-type strain, which was used for the sequencing of the P. anserina genome (2, 11, 50). The two culture media used for this study are the most commonly used M2 medium (0.25 g/L KH_2_PO_4_, 0.3 g/L K_2_HPO_4_, 0.25 g/L MgSO_4_, 0.5 g/L urea, 0.05 mg/L thiamine, 0.05 μg/L biotine, 5 mg/L citric acid, 5 mg/L ZnSO_4_, 0.25 mg/L CuSO_4_, 50 μg/L MnSO_4_, 50 μg/L boric acid, 50 μg/L natrium molybdate, 1 mg/L iron alum, 5.5 g/L dextrin, and 10 g/L agar) with pH maintenance at 7 by a phosphate buffer and the G (germination) medium (25 g/L corn flour, 25 g/L corn cream, 6 g/L ammonium acetate, and 12g/L agar).

All the protocols, including standard culture conditions and genetic manipulation for this microorganism, are described by Silar (4) and can be accessed at http://podospora.i2bc.paris-saclay.fr. The *Δmus51*::*nourR* strain lacking a *mus51* gene was used for deletion mutant construction, which resulted in an increased frequency of targeted gene replacement (51). The *ΔPaStcA*::*genetR* strain harboring a mutation in the PKS-encoding gene *PaStcA*, which acts at the first step of ST biosynthesis, was previously constructed by Shen et al. (14).

### Deletion and complementation of *PaNsdD*.

The *PaNsdD* (*Pa_2_1880*) gene sequence was retrieved from the P. anserina genome database by BLAST, using the N. crassa protein SUB-1 (NCU01154) as query (11). To investigate the function of PaNsdD, targeted gene deletion was carried out according to the “split marker” method, as previously described (4, 52). Briefly, a phleomycin resistance cassette was used as selective marker. The flanking regions (approximately 800 bp) of the open reading frame of *PaNsdD* were amplified and then were fused with the resistance cassette, respectively. The fusion products were then used to transform protoplasts of the *Δmus51*::*nourR* strain. All transformants were screened on minimal medium containing phleomycin at 10 μg/mL. Putative transformants were then diagnosed by PCR. The primers used in this study are listed in Table 2. To eliminate the untransformed nuclei and to segregate the *Δmus51* mutation, two primary transformants *ΔPaNsdDΔmus51* (PhleoR HygroR) were genetically purified by crossing with the wild-type strain to generate both *mat+* and *mat*− homokaryotic mutant strains *ΔPaNsdD* (PhleoR HygroS) that contain only the *PaNsdD* deletion but lack *Δmus51*. Correct gene replacement was further confirmed by Southern blotting (Fig. S1). The *ΔPaNsdD* mutant was genetically crossed with *ΔPaStcA* to generate the double mutant *ΔPaNsdDΔPaStcA*, which was resistant to both phleomycin and Geneticin.

**TABLE 2:** Primers used in this study

Primers	Sequence (5′ to 3′)	Application
PaNsdD_1F	tccagattggcaggacagtg	Deletion of *Pa_2_1880*
PaNsdD_2R	ctatttaacgaccctgccctgaaccgggacttgggaccgtttttgg	
PaNsdD_2F	ccaaaaacggtcccaagtcccggttcagggcagggtcgttaaatag	
PaNsdD_3R	ggtcaatactagctccggtcccatcgaactggatctcaacagcggtaag	
PaNsdD_3F	cttaccgctgttgagatccagttcgatgggaccggagctagtattgacc	
PaNsdD_4R	actaaaggtgcccaaacttgc	
PaNsdD_verify_F	cgaattgttccgccttttcg	Junction verification for *Pa_2_1880* deletion
Valid5’	tgagaagcacacggtcac	
Valid3’	tcggggcgaaaactctc	
PaNsdD_verify_R	tgaatccgagcctctgttgtg	
PaNsdD_cF	attggcaggacagtgacatc	Complementation construct of *Pa_2_1880*
PaNsdD_cR	gtctttcaggcgagaacagc	
PaNsdD_verify_cF	ccagtgcctcaacaaatgcc	Verification for *Pa_2_1880* complementation
PaNsdD_verify_cR	gctgttttcctcaccatacg	

Complemented strains were generated by ectopically introducing a wild-type allele of *PaNsdD* into corresponding mutant, as described by Shen et al. (14). To obtain the plasmid pBC-hygro-PaNsd-Comp, a wild-type *PaNsdD* gene containing native promoter and terminator was amplified with primers PaNsdD_cF and PaNsdD_cR (Table S1). Fragment was inserted into EcoRV-linearized pBC-hygro plasmid. The reconstructed vector was validated by sequencing (Genewiz, Germany) and then was transformed into the *ΔPaNsdD* mutant strain. All hygromycin-resistant transformants were verified by PCR and phenotypic analysis; three out of putative transformants that restored the wild-type-like phenotype were genetically purified, resulting in the complemented strain *ΔPaNsdD^C^*, which was selected for detailed phenotypic characterization.

### Phenotypic analysis.

To determine the role of PaNsdD in fungal physiology, wild-type and mutant strains were cultured on M2 medium at 27°C. Aliquots (5 μL) of standardized fragmented mycelial suspension were spotted on the plates. For vegetative growth observation, colony morphology, size, and pigmentation were documented each day. For fertility assessment, perithecium formation, ascospore production, and dispersal were examined during their sexual cycle. In order to observe the fungal pigmentation of thallus and the coloration of culture filtrate, M2 and LB liquid cultivation were also similarly conducted. All experiments were performed in triplicate.

### Microscopy analysis and quantitative measurement of spermatia.

Microscopic observation was made on fresh mycelia growing on solid M2 medium. Quantitative measurement of spermatia was conducted as described previously (53). Briefly, the strains were cultured for 3 days, and then the spermatia were harvested by flooding the plates with 1.5 mL of 0.05% Tween 20 in sterile water. The suspension was counted through a hemacytometer.

### Sensitivity to various abiotic stresses.

To assess the sensitivity of fungal cells to environmental stresses, fungal growth was determined on M2 medium supplemented with various chemical stressors at a certain concentration: (i) H_2_O_2_ (500 μM), menadione (Mena, 25 μM), *t*-butyl hydroxyperoxide (TBY, 50 μM), or methylglyoxal (MG, 5 mM) for oxidative stresses; (ii) Sorbitol (1.5 M) for osmotic stress, and (iii) Congo red (CR, 100 μM) or Calcofluor white (CFW, 10 μM) for cell wall-perturbing stresses. Aliquots (5 μL) of standardized fragmented mycelial suspension were spotted on the plates. Colony diameters were measured daily from three replicates. Quantification of relative growth inhibition of strains exposed to different chemicals was calculated as *D_t_*/*D_c_* (where *D_c_* is diameter of control colony without treatment, and *D_t_* is the diameter of colony treated with stressors).

### Interspecific confrontation assay.

Detection of cell death and oxidative burst were conducted as previously described (14, 32). Briefly, P. anserina wild-type and mutant strains were inoculated on M2 plates with three neighboring P. chrysogenum strains and then incubated for 3 days. T. versicolor and B. cinerea strains were centrally precultured on M2 plates for 4 days, prior to the inoculation of P. anserina wild-type and mutant strains. Cocultivation can be ceased until the central fungus was completely in contact with competing fungal thalli (14). Trypan blue staining was used to reveal the cell death in the contact zone (32). Diaminobenzidine (DAB) and nitroblue tetrazolium (NBT) staining were used to detect peroxide and superoxide, respectively (54, 55).

### Metabolite extraction and analysis.

To monitor the production of putative compounds regulated by PaNsdD, P. anserina wild-type and mutant strains were stationarily cultured on M2 and LB liquid medium at 27°C for 14 days. Secondary metabolite extraction was conducted as described previously (14). The extracts were dissolved in 2 mL of methanol, filtered over a 0.2-μm filter, and subjected to reversed-phase high performance liquid chromatography coupled to a diode-array detector (DAD) analysis. HPLC analysis was performed on Dionex UltiMate 3000 HPLC Systems using a column (X-bridge C18, 4.6 × 250 mm, 5 μm, Waters, Ireland) with a flow rate of 1 mL/min. Fresh extracts of all strains were detected with a gradient of acetonitrile in H_2_O: 0% for 5 min, 0 to 100% in 35 min, and 100% for 10 min.

In order to identify peaks detected on HPLC-UV chromatogram, ST standard previously isolated was used (14). Concerning the peak at 16.05 min, which disappeared in the *ΔPaNsdD* strain, its purification was conducted as follows. The extract (125 mg) was purified by chromatography on silica (20 to 45 μm) with a gradient of ethyl acetate in cyclohexane (0 to 100%) to give a fraction of 4.5 mg containing the unknown compound. A subsequent purification was made on a preparative HPLC system (Shimadzu, Kyoto, Japan) consisting of an LC-40 delivery system, Rheodyne manual injection valve, FRC-40 fraction collector, and SPD-M40 detector connected to a SCL-40 control unit. The fraction was dissolved in 0.5 mL of MeOH and purified on a Nucleodur C18 Htec (250 mm × 32 mm, 5 μm) column (Macherey-Nagel) with a gradient of acetonitrile in water (10 to 80% for 30 min) at a flow rate of 45 mL/min. Fractions containing the products were pooled, and the mobile phase was evaporated under reduced pressure to furnish 1.9 mg of the desired pure compound. NMR and MS data of this metabolite were in accordance with the literature (33, 34) and were permitted to identify the product as 3-acetyl-4-methylpyrrole.

### Data availability.

All of the data generated in this study are included in the main text and its supplementary information files.
