# Supplementary material for: Functional Characterization of the GATA-Type Transcription Factor PaNsdD in the Filamentous Fungus Podospora anserina and Its Interplay with the Sterigmatocystin Pathway
Source: Appl Environ Microbiol. 2022 Mar 22;88(6):e02378-21. doi: 10.1128/aem.02378-21 (PMC8939327; doi:10.1128/aem.02378-21)

Figure S1. validation of deletion mutants and complemented strains.

(A) Genomic DNA was isolated from wild type (WT) and two independent deletion transformants, and was then digested with the indicated restriction enzyme *Xho* I. The blots were probed either with a sequence containing the relevant flanking region. A restriction map of the WT and mutant locus is presented. (B) The sizes of the expected fragments are indicated on the maps and are reported close to the corresponding fragment on the southern blot. (C) Primers used for deletion construction and junction verification are also indicated. PCR screening for gene deletion of putative independent transformants were performed using for the primer pairs of verify-F/valid-5' (left panel) and verify-R/valid-3' (right panel). (D) PCR verification for complementation using the primer pairs (verify-cF / verify-cR). The *ΔPaNsdD* mutant was used as negative control.

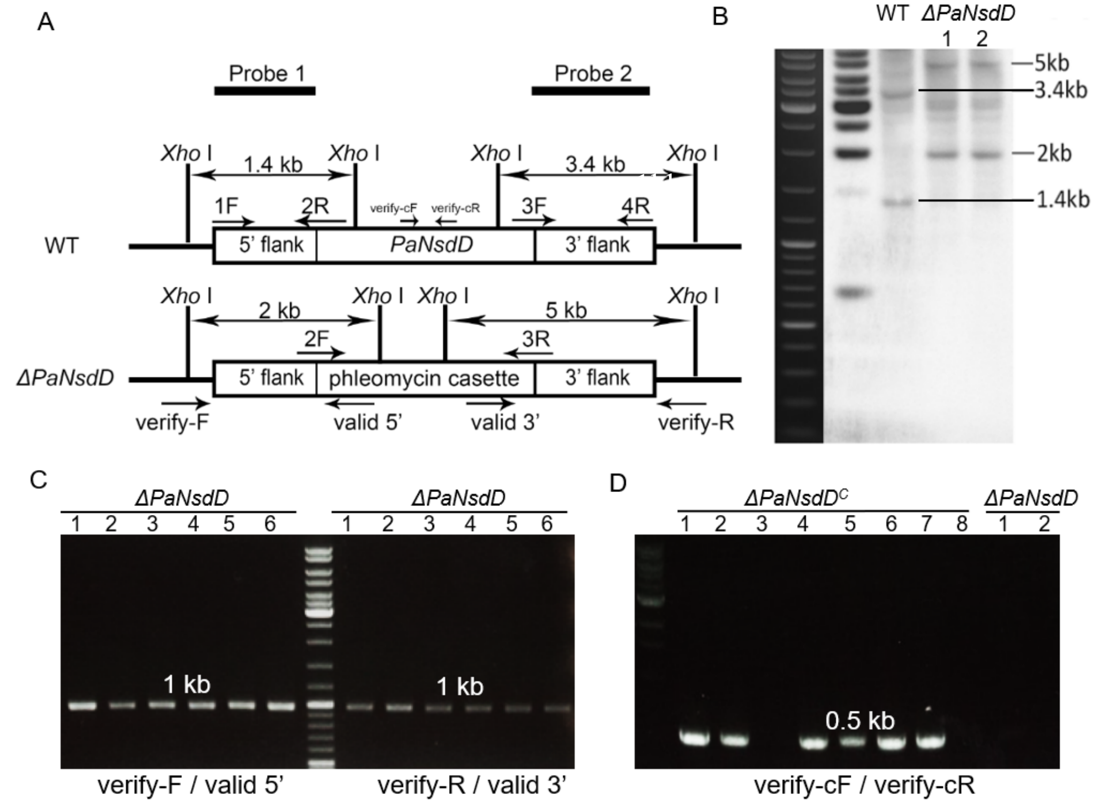

Figure S2. Peroxide detection of *P. anserina* strains when contestants are absents. Detection of oxidative burst were conducted as described in experimental procedures. *P. anserina* wild-type and mutant strains were inoculated on M2 plates and then incubated for 3 days. Diaminobenzidine (DAB) staining was used to detect peroxide respectively.

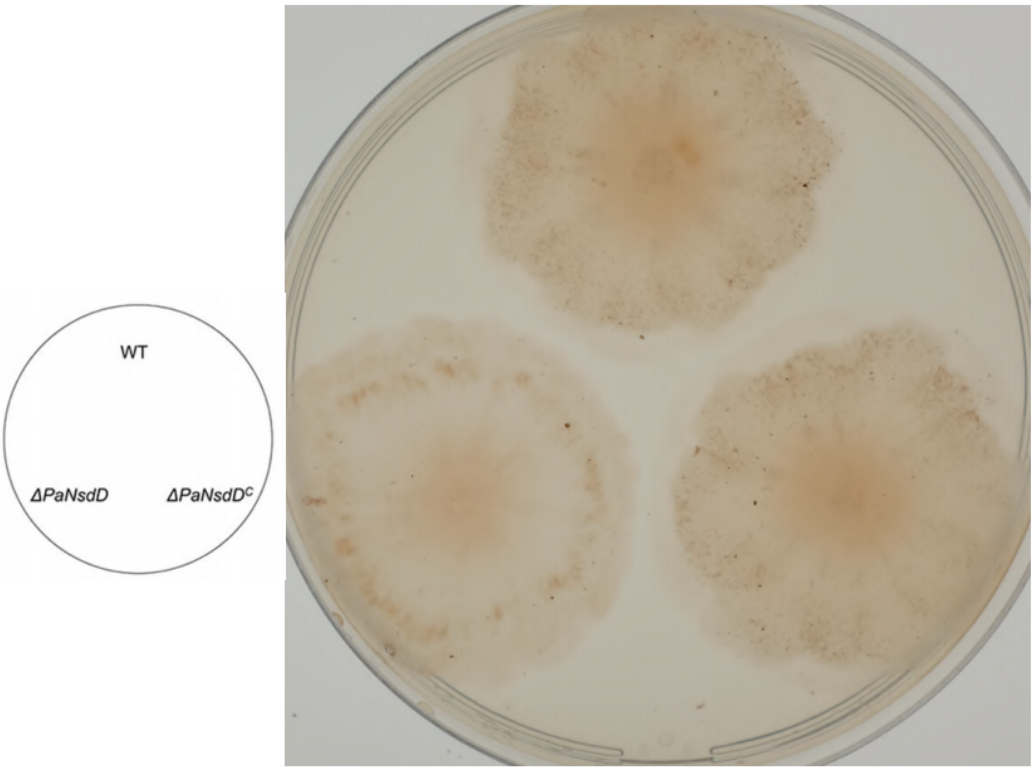

Figure S3. Growth of *P. anserina* strains on G medium and G+yeast medium.

The wild-type, *ΔPaNsdD*, *ΔPaStcA* and *ΔPaNsdDΔPaStcA* strains were grown on G and G+yeast solid medium at 27 °C under dark condition. Pictures were taken after eight days of incubation as indicated. WT: wild-type strain.

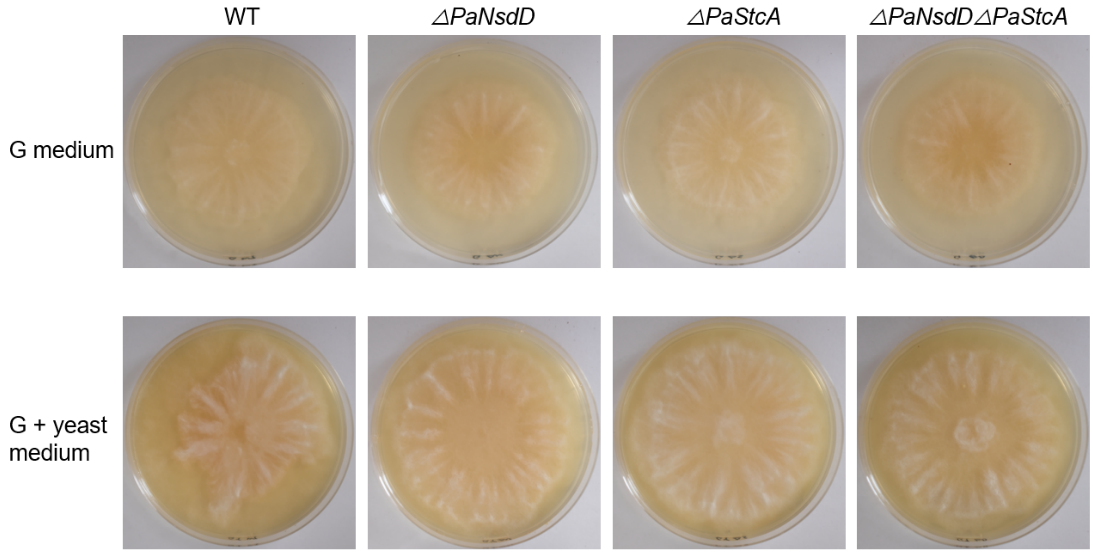

Figure S4. HPLC-UV detection of LB culture extracts of *P. anserina*.

The HPLC-UV profiling displayed the peak intensity (mAU) according to the retention time (rt). ST presented a rt of 27.3 min. WT: wild type strain. X-axis (min): retention time. Y-axis (mAU): signal intensity.

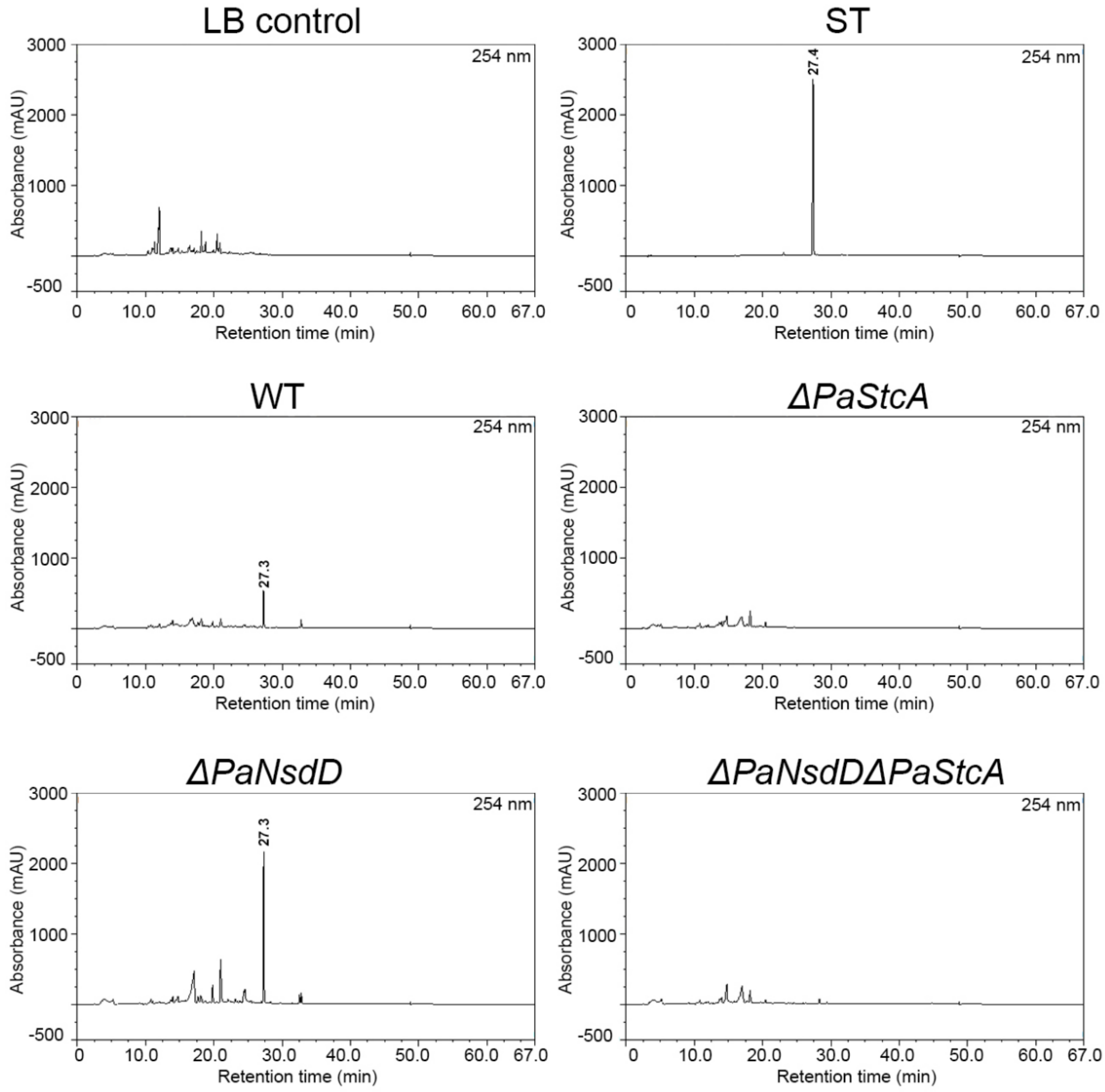

Figure S5. LC/MS analysis.

The isolated product corresponding to the peak at 16.05 min was conducted by LC-MS in positive ESI and negative ESI mode, respectively.

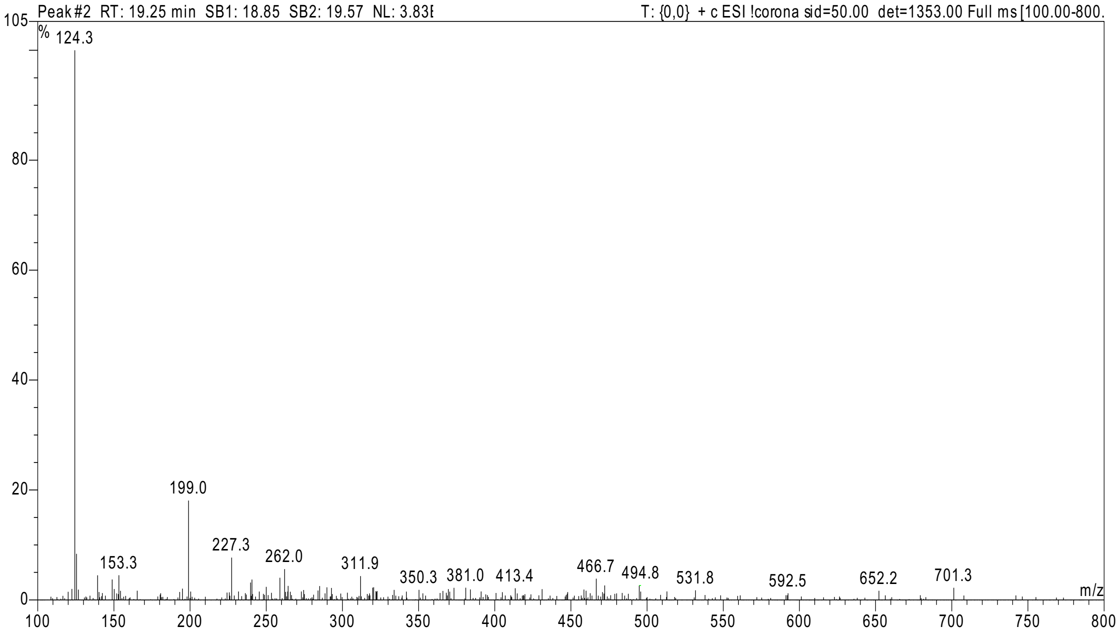

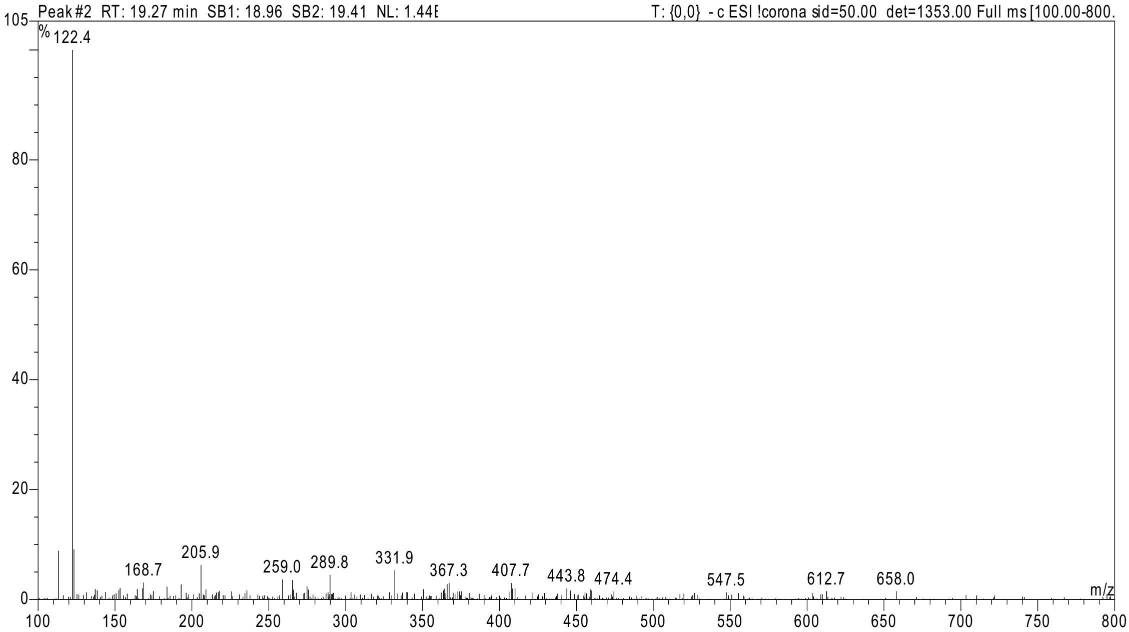

Figure S6. NMR analysis.

^{1}H and ^{13}C NMR spectra of the isolated product corresponding to the peak at 16.05 min (solvent $CDCl_3$).

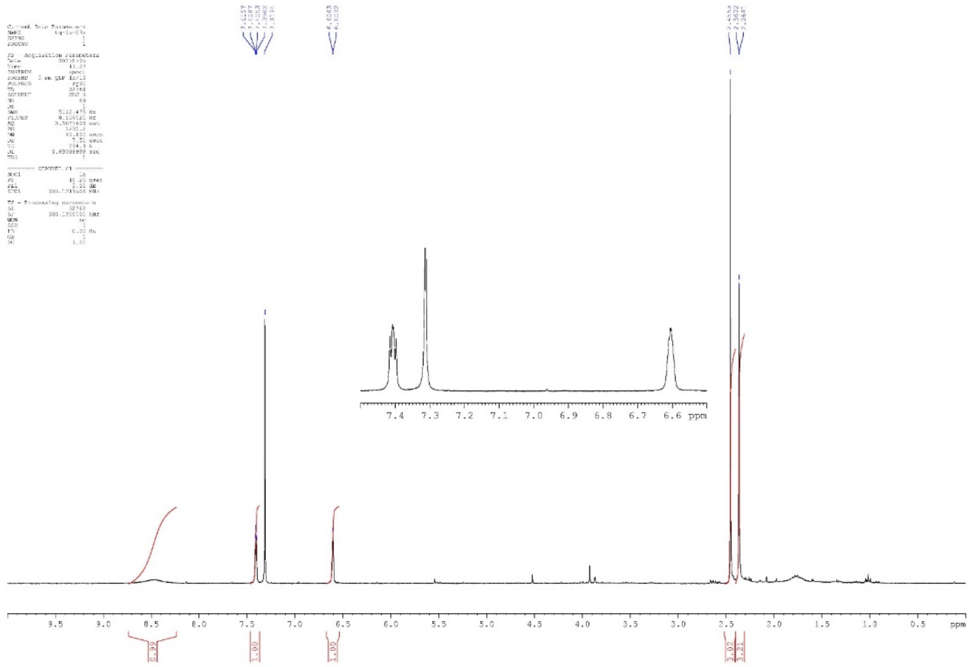

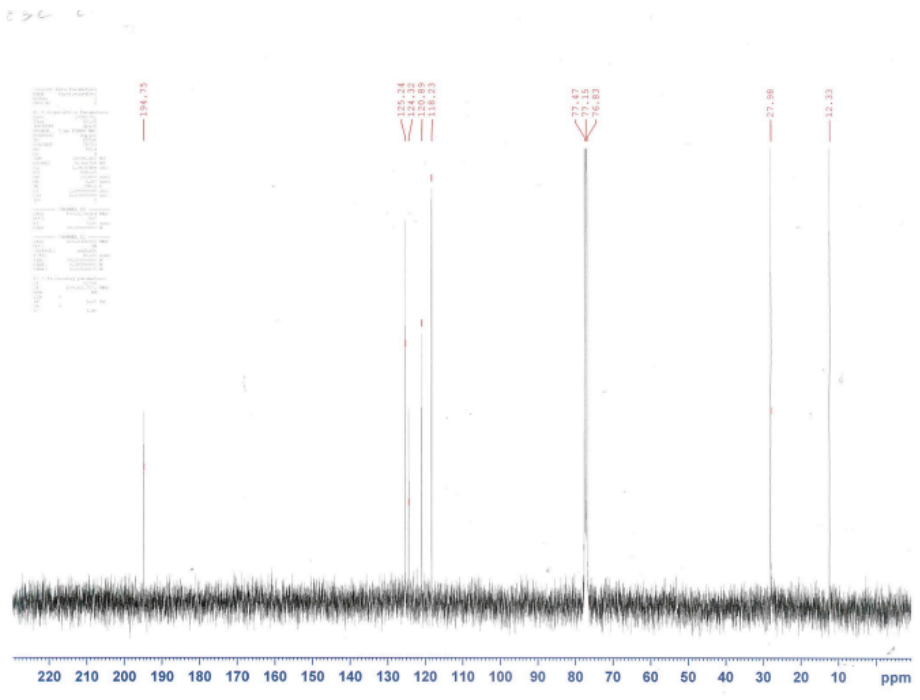

Supplement: Supplemental file 1 — Fig. S1 to S6. Download aem.02378-21-s0001.pdf, PDF file, 2.4 MB [file aem.02378-21-s0001.pdf]
